# Mining and modelling temporal dynamics of followers’ engagement on online social networks

**DOI:** 10.1007/s13278-022-00928-2

**Published:** 2022-07-31

**Authors:** Luca Vassio, Michele Garetto, Emilio Leonardi, Carla Fabiana Chiasserini

**Affiliations:** 1 Politecnico di Torino, Torino, Italy; 2 University of Turin, Torino, Italy; 3 CNIT, Parma, Italy

**Keywords:** Online social networks, Temporal dynamics, Popularity evolution, User engagement, Facebook, Instagram

## Abstract

A relevant fraction of human interactions occurs on online social networks. In this context, the freshness of content plays an important role, with content popularity rapidly vanishing over time. We therefore investigate how influencers’ generated content (i.e., posts) attracts interactions, measured by the number of likes or reactions. We analyse the activity of influencers and followers over more than 5 years, focusing on two popular social networks: Facebook and Instagram, including more than 13 billion interactions and about 4 million posts. We investigate the influencers’ and followers’ behaviour over time, characterising the arrival process of interactions during the lifetime of posts, which are typically short-lived. After finding the factors playing a crucial role in the post popularity dynamics, we propose an analytical model for the user interactions. We tune the parameters of the model based on the past behaviour observed for each given influencer, discovering that fitted parameters are pretty similar across different influencers and social networks. We validate our model using experimental data and effectively apply the model to perform early prediction of post popularity, showing considerable improvements over a simpler baseline.

## Introduction

Billions of people use online social media applications such as Facebook (FB) and Instagram (IG) as part of their daily activities. Social media applications indeed make possible to exchange opinions, get news and maintain social interactions through posts, comments, and likes. In particular, FB has been the most popular social media application for quite a long time, while IG has experienced a surge in popularity in the last few years. In both Facebook and Instagram, *influencers* (i.e., popular users, groups, newspapers, or companies) post content (i.e., the so-called *posts*) in the form of photos, videos or texts. Users of these social networks can *follow* influencers and interact with posts by *liking*, *reacting*, *sharing*, or *commenting* them.

Several studies on online social networks (OSN) have analysed content popularity as a function of the total number of interactions (views, likes, etc.), measured at the time data was crawled. Many works focus on predicting the popularity of posts, often given their intrinsic characteristics as well as the characteristics of the influencers and their followers. Few works, instead, focus on understanding the temporal dynamics of the popularity of content generated in OSNs. While it has been largely recognised that content popularity decreases over time, different models have been proposed for the decay rate of popularity, depending on the platform and content itself. Sometimes popularity is modelled by a negative exponential function, sometimes by heavy-tailed functions, and in other cases simply as constant (see Sect. 2).

However, a large-scale characterisation of the temporal evolution of the popularity of posts in OSNs is still missing. In this work, we aim at filling this gap by (i) providing an experimental analysis of the time evolution of interactions with user-generated content, both on a per-post and per-influencer basis, and (ii) developing an analytical model capturing the main aspects of user interactions on OSNs.

To this end, we focus on two popular social networks, Facebook and Instagram. These applications currently have a large ecosystem of influencers that try to gain popularity in different ways, e.g., by increasing the number of posts, by posting content of large or mixed interest, by debating or posting a reply on others’ posts (Kim et al. 2017). In this work, we analyse, model, and compare user engagement and interactions by leveraging a dataset of more than 13 billion interactions over approximately 4 million posts of 651 Italian influencers on FB and IG. The collected dataset covers a period of more than 5 years, from January 1, 2016 to June 1, 2021.

We analyse such data aiming at answering the following fundamental questions. *What are the main factors impacting on temporal dynamics of posts published by the influencers? How do followers interact with such posts? In particular, what is the time evolution of the reactions to these posts? Can we develop a model of these dynamics and exploit it for practical applications?*

Our main findings can be summarised as follows:

- Both influencers’ activity and users’ activity exhibit a characteristic daily pattern, but with a different shape;
- The inter-arrival time of posts has a long-tail distribution, reasonably fit by a log-normal;
- On average 50% of user interactions occur within the first 4 h after content creation on FB, and after 2 h on IG; interactions arrival rate exhibits approximately an exponential temporal decay;
- Most of the posts are short-lived, with a lifetime between 20 and 50 h, after which they no longer attract interactions;
- The fraction of total interactions obtained within a given time interval is affected by the number of newly published posts in the same interval;
- The distribution of the total number of interactions (likes, reactions, comments, and shares) is well fit by a log-normal distribution;
- The average number of interactions received by posts is roughly linear with the number of followers of the publishing influencer;
- The total number of interactions gathered by a post can be well predicted by measuring the interactions received within the first hour or even from the first few minutes.

Our exploratory data analysis identifies the main features that should be incorporated into an analytical model trying to capture the temporal evolution of interactions received by a post. We first attempt to develop such a model, fitting a small set of parameters to the specificity of posts published by a given influencer. Interestingly, we discover that many of these parameters do not vary significantly from influencer to influencer; moreover, they only weakly depend on the considered social network (IG or FB).

Our model can provide an accurate prediction of the total number of interactions gathered by a post (and an estimate of the prediction error) by observing only the initial phase of its lifetime. We believe this ability of our model can have interesting applications. Finally, we mention that a preliminary version of our work, presenting a subset of the results obtained from our dataset, has appeared in Vassio et al. (2021). This paper extends the data analysis and introduces a novel model for the temporal evolution of interactions with posts, which is then validated and applied to early prediction of post popularity.

The remainder of the paper is organised as follows. Section 2 summarises some relevant related work. Section 3 describes the methodology we used to extract and process the data, while Sect. 4 presents the results of our data analysis. Section 5 describes the complete analytical model that we have developed, which is then evaluated and compared to a baseline. Finally, Sect. 6 concludes the paper.

## Related work

Social media provide a powerful and effective platform for the exchange of ideas and rapid propagation of information (Al-Garadi et al. 2018). Hence, their study is of paramount importance to understanding the opinion trends in our society and the main actors, i.e., the influencers (Conover et al. 2012; Gorkovenko and Taylor 2017; Pierri et al. 2020).

Although a large body of literature has analysed OSNs, the temporal dynamics of posts and interactions are still not well understood. Indeed, the majority of existing studies ignore such temporal dynamics, focusing on the “spatial” analysis of a single, large snapshot. A few works have focused on predicting content popularity, considering content intrinsic characteristics and social interactions features (Li et al. 2013; Rizos et al. 2016). The main factors that impact the popularity of posts on FB are identified in Sabate et al. (2014), using an empirical analysis involving multiple linear regressions. Similarly, (Ferrara et al. 2014) highlights the characteristics related to the dynamics of content production and consumption in IG, while (Gayberi and Oguducu 2019) and (Carta et al. 2020) predict the popularity of a future post on IG by combining user and post features.

Instead, few studies have analysed the time dynamics of content generated in OSNs. The decay in popularity over time, i.e., the rate of new interactions, of Internet memes (Leskovec et al. 2009) is shown to be well modelled by a negative exponential function. The work in van Zwol (2007) measures the time evolution of the popularity of images in Flickr, finding that heavy-tailed distributions can represent the decay in rate of new interactions over time. Instead, (Cha et al. 2009b) observe that the most popular Flickr pictures exhibit a close-to-constant interaction rate. The study in Hassan Zadeh and Sharda (2014) models the popularity evolution of posts by Hawkes point processes, using Twitter data to fit the required parameters. Gabielkov et al. (2016) analyse and predict clicks on Twitter posts. They find that while posts appear as bursts in a short-time frame, clicks appear and decay at larger time scales, with a long tail. The authors leverage early interactions to predict future clicks, as in our work. They show that a simple linear regression based on the number of clicks received by tweets during its first hour correctly predict its clicks at the end of the day, with a Pearson correlation of 0.83. Ramachandran et al. (2018) propose a model that reproduces the clicks created by social media. In particular, the authors consider news posted on Twitter, and observe that hourly impressions decrease geometrically with time. They model information diffusion to determine current and future clicks, using a memoryless generative model with a few time-invariant parameters. Finally, Ferrara et al. (2014) show that the distribution of likes to posts on IG is best fit by a power-law, suggesting that popularity of media as measured by the number of likes might grow by a preferential attachment mechanism. However, Ferrara et al. (2014) provide no evidence of this kind of evolution.

Other works analyse the temporal dynamics of particular user-generated content, outside of OSNs. For example, videos on YouTube exhibit various popularity decay patterns over time (Cha et al. 2009). For some videos, the decay can be modelled with heavy tail distributions, while for others with an exponential distribution. Similarly, Ahmed et al. (2013) show that user generated videos have distinct patterns of popularity growth (in terms of views) over time.

Our previous studies (Ferreira et al. 2020; Ferreira et al. 2021) focus on the peculiarity of user interactions with political profiles on IG during the 2018 European and Brazilian elections, with the goal of identifying the structure emerging from the co-interactions. We studied the appearance and evolution of communities of users, obtained through a probabilistic model that extracts the backbone of the interaction networks. Interestingly, politicians are able to attract more persistent communities over time than non-politicians. Related to the topic of popularity prediction, we proposed (Bertone et al. 2021) a parallel between the OSN world and the stock market: influencers can be viewed as stocks while users are investors. The study shows how this market-like approach successfully estimates short-term trends in influencers’ followers from external variables, such as Google Trends. Finally, our previous study (Trevisan et al. 2021) investigates the changes in habits in OSNs during the COVID-19 outbreak. It is shown how people, during the lockdown, due to restrictions enforced to in-presence social activities, changed their interaction patterns, shifting more towards the night.

We emphasise that a large-scale characterisation of the temporal evolution of post popularity in OSNs is still missing. In this work, we aim to fill this gap by providing (i) an experimental analysis of the time evolution of interactions with user-generated content, both on a per-post and per-influencer basis, and (ii) an analytical model that can accurately represent user interactions on OSNs.

## Data collection

**Table 1:** Features of the influencers dataset we built, as recorded in June 2021

	Influencers	Avg followers	Posts	Interactions	Comments	Shares
Instagram	244	1.19 mil.	0.31 mil.	9.36 bil.	0.12 bil.	NA$^{a}$
Facebook	407	1.55 mil.	3.57 mil.	4.02 bil.	0.63 bil.	1.32 bil.

$^{a}$ On Instagram it is not possible to share/repost a post on the feed

In FB and IG, a *profile* can be followed by other profiles, i.e., its *followers*. A profile with a large number of followers is also called an *influencer*. Influencers post content (i.e., *posts*), consisting of either a photo or a video, or plain text. The profile’s followers, and anyone registered on the platform in the case of public profiles, can *view* the influencer’s posts, *like*/*react* to them, *comment* on them, and *share* them with their contacts. Notice that, by the term influencer, we refer not only to individuals, but also to groups, football teams, newspaper, and companies.

We monitored the activities triggered by top Italian influencers on the two aforementioned social networks. To this end, we built lists of the most popular Italian influencers, including different categories, like politicians, musicians, and athletes. Those marked as Italian are the ones that communicate on the online social platform mainly using the Italian language.

To get popular profiles, we exploited the online analytics platform www.hypeauditor.com for IG, and www.socialbakers.com and www.pubblicodelirio.it for FB. The analysis has been restricted to the influencers with at least 10, 000 followers on June 1, 2021. The lists of influencers we used are publicly available.^1^

For each monitored profile, we downloaded the corresponding metadata, i.e., the profile information, and all the generated posts, using the CrowdTangle tool and its API.^2^ CrowdTangle is a content discovery and social analytics tool owned by Meta,^3^ which is open to researchers and analysts worldwide to support research, upon subscription of a partnership agreement. Furthermore, for each post, we downloaded the number of associated interactions, along with their timestamp. Monitored posts are sampled by CrowdTangle within the first 20 days (480 h), with a higher sampling rate (up to few minutes) closer to the publication time of the post. Notice that, on IG, users can *like* posts, whereas on FB, they can *react* to posts with a thumbs up or other five pre-defined emojis. Thus, for each post, we collected the *number of likes/reactions* the post received, hereinafter referred to as *interactions*, which CrowdTangle provide in an anonymized manner. Moreover, we also collect statistics about number of comments per post for FB and IG and number of times posts are shared for FB. Finally, we have stored the data, which takes around 110 GB of disk space, on a Hadoop-based cluster, and we have used PySpark for scalable processing.

For each influencer, we downloaded all the data related to the posts published between January 1, 2016 and June 1, 2021. Table 1 reports the main features of our dataset, separately for each OSN. In total, we monitored 651 public profiles, which published approximately 4 million posts, accounting for more than 13 billion interactions. The number of comments and shares of the posts are also reported. Notice that while the influencer’s posts are widely shared by their followers (around 1.3 billion times, hence on average 370 times per post), our analysed influencers rather rarely repost other influencers’ posts. Indeed, we observed only around 24 thousand shared posts by the influencers on FB, accounting for only about 0.7% of all the posts.

**Fig. 1:**
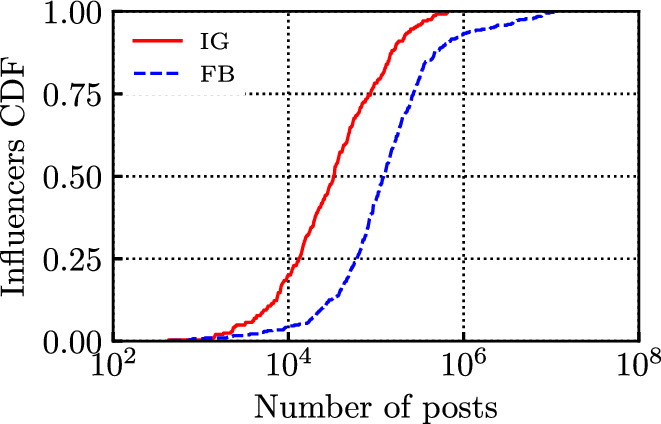
CDF of the number of posts for the considered influencers

**Fig. 2:**
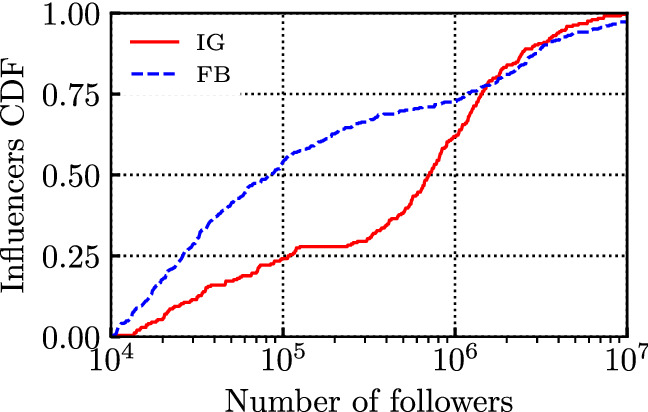
CDF of the number of followers for the considered influencers, in June 2021

Figure 1 depicts the empirical Cumulative Distribution Function (CDF) of the number of posts per influencer. The 651 influencers show a large variability in the distribution of number of posts: some influencers published few tens of posts, while others, such as newspapers pages, up to $10^5$ posts. Also, in the period under study, influencers on FB published more than those on IG. The main reasons are twofold: i) on FB more influencers are actually pages or organisations, rather than single individuals, and ii) many popular IG influencers did not exist at the beginning of the considered time period (i.e., in 2016), or have become active much later. Figure 2 depicts the Cumulative Distribution Function (CDF) of the number of followers per influencer, as recorded on June 1, 2021. The number of followers per influencer varies between 10k and tens of millions. Also, the profiles in the set chosen for IG are usually more popular than those selected for FB.

## Temporal user engagement with posts

In this section, first we characterise the patterns of the influencers’ and followers’ activity (Sect. 4.1), then we study the time evolution of interactions (Sect. 4.2) and their relation with the number of followers (Sect. 4.3). Finally, we investigate the correlation between the interactions a post attracts and the number of newly published posts (Sect. 4.4).

### Activity of influencers and followers

**Fig. 3:**
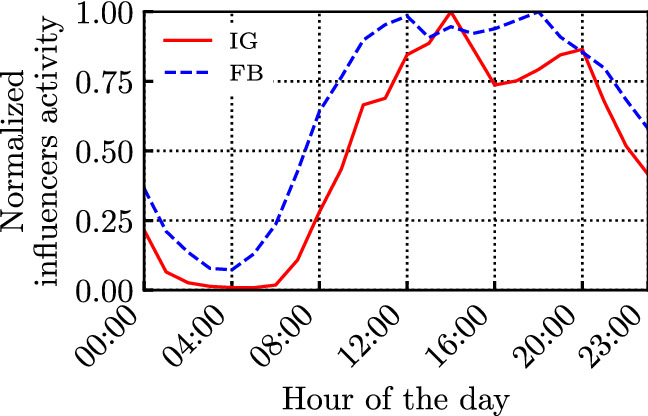
Normalized daily activity (i.e., creation of posts) of the sampled influencers

**Fig. 4:**
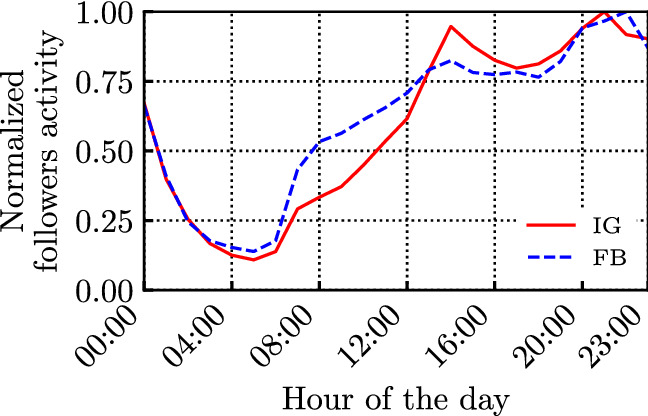
Normalized daily followers’ activity (i.e., their interactions with the influencers)

**Fig. 5:**
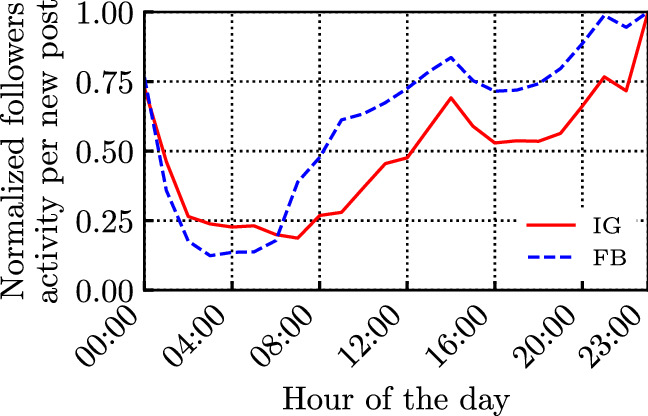
Normalized daily followers’ activity (i.e., their interactions) per new post by the influencers

We first characterise the daily patterns of influencers’ and followers’ activity. Figure 3 presents the influencers’ hourly activity, obtained considering the time instants at which posts were published. The activity is normalised by their maximum in both social networks to have comparable results. The plot accounts for all the analysed 4 million posts, and it is reported using a 24-hour local-time clock (using the Italian time zone), according to the ISO 8601 standard.

Similarly, Fig. 4 shows the daily activity distribution of the followers, considering the timestamps of the followers’ interactions (considering all 13 billion likes/reaction). Note that, due to our particular selection of influencers, we can reasonably expect that the vast majority of posts and interactions occur in the same time zone (the Italian time-zone). This is also supported by the results in Benevenuto et al. (2012), where authors show that followers/friends interacting in social networks are usually within close geographical proximity of the influencer.

We observe that influencers’ and followers’ activities exhibit similar patterns over FB and IG: they significantly decrease during the night, and exhibit two peaks during the day. However, it is interesting to notice that followers tend to be more active later in the evening with respect to influencers.

Moreover, looking at the behaviour of specific influencers on FB and IG (the results are omitted for brevity), we observed that their followers’ activity over time tend to be similar to that in Fig. 4, although the single influencer’s daily activity might deviate significantly from the one shown in Fig. 3. This is confirmed by the results in Fig. 5, showing that the average followers’ activity per new post maintains a similar shape to the ones in Fig. 4. Although influencers generate very few posts late at night, such posts are typically fresher and encounter less ‘competition’. Nonetheless, they still collect very few interactions during the night.

**Fig. 6:**
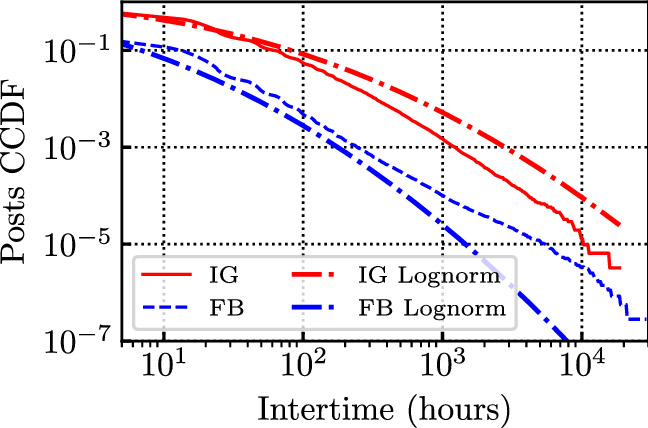
CCDF of the intertime of the posts produced by the influencers (loglog scale), along with their log-normal fitting

We now investigate the distribution of the inter-arrival time between different posts. In particular, we focus on the tail of the distribution, considering time-scales of several tens of hours, i.e., time-scales at which the impact of the day-night activity pattern is negligible. Figure 6 depicts the tail of the intertime of all posts generated by the influencers, including the best fitting log-normal distribution. The log scales in the plot suggest that the log-normal distribution provides a substantially better fit than what would be obtained by an exponential distribution (i.e., under a Poisson process assumption). This is due to the fact that influencers sometimes remain silent for (quite) long periods. We also analysed single influencers and found that, for the median influencer, the average posts inter-arrival time is equal to 19 hours on FB, and 57 hours on IG. Then, fitting separately each influencer with a log-normal distribution, on average, we obtained as parameters of the log-normal $\mu =2.0$, $\sigma =1.4$ on FB, and $\mu =3.1$, $\sigma =1.3$ on IG.

### Temporal dynamics of interactions

**Fig. 7:**
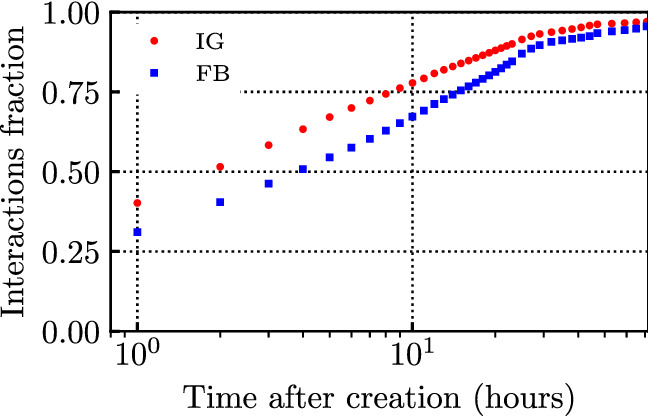
Average evolution of a post in terms of interactions, over 3 days (72 h)

**Fig. 8:**
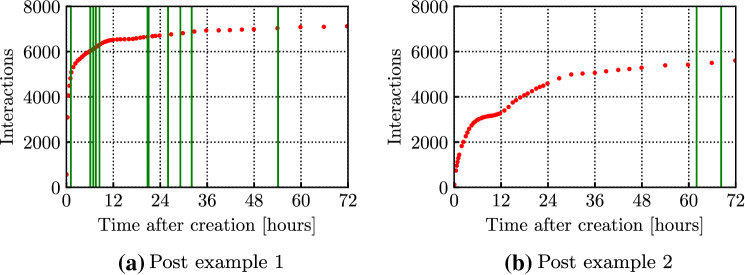
Two examples of the evolution over 3 days of the interactions to a post. Newly published post timestamps are highlighted with vertical lines

We now analyse the temporal evolution of the interactions to a post, considering up to 20 days (480 h) after the creation of the post itself. We compute, for all the 4 million posts and for every sample-time (rounded to the closest integer hour), the fraction of received interactions with respect to the total number of interactions obtained by a post after 20 days. We consider fractions in order to compare different posts, and different influencers. Finally, we compute the average over all posts.

The results, representing the dynamics of the average fraction of interactions over the first 3 days since the creation of the post, are shown in Fig. 7. One can notice that the majority of the interactions occur within the first few hours. On average, the first hour accounts for 31% of all of the interactions on FB (40% on IG), reaching over 80% after 1 day. Moreover, on average, 50% of user interactions occur within the first 4 h since content creation on FB, and after 2 h on IG. It is thus clear that the freshness of a post has a significant impact on the level of attractiveness of the post. Interestingly, the growth of the number of user interactions is faster on IG than on FB, although both curves converge after around 30 h. Studying the evolution of the rate of new interactions, we found that, at least in the first 24 h after the post creation, this rate is well approximated by a negative exponential decay function (with mean equal to 5.4 for FB, and 8.7 for IG).

As expected, individual posts can have widely different patterns in terms of their accumulation of interactions over time. As an example, we show the results related to two specific posts on FB published by a well-known Italian influencer (namely, Giuseppe Conte, former Italian Prime Minister). The temporal dynamics of interactions over the first 3 days since the post creation are represented by red marks in Fig. 8a and 8b. We notice the presence of periods in which the number of interactions is almost constant, after which it increases again. We verified that this behaviour is essentially due to the non-stationary behaviour of users’ activity during the day (see Fig. 4 and 5), i.e., quasi-flat portions of the curves correspond to night hours. Green vertical lines highlight newly published posts (see Sect. 4.4). For the first example trace (left plot), many posts are published within the first three days; for the second trace (right plot), no new post is published before the first 62 h.

**Fig. 9:**
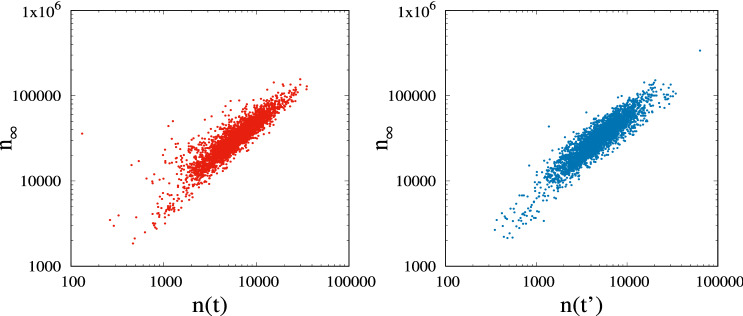
Total number of interactions $n_\infty$ vs. number of interactions received after 30 min, considering physical time (left plot) and virtual time (right plot)

We now turn to the interesting question of whether the total number of interactions collected by a post can be forecast by observing just the interactions received during an initial interval after publication. A first, strong indication that such prediction is indeed feasible is illustrated in Fig. 9, showing a scatterplot of roughly 3,000 points, each corresponding to a post published on IG by a given influencer (in this case, the Italian politician Matteo Salvini): the *y* axes provide the total number of interactions, while the *x* axes correspond to the number of interactions received after half an hour.^4^

The left plot corresponds to measurements *n*(*t*) collected at physical time, while the right plot correspond to measurements $n(t')$ transformed into virtual time to remove daily effects (see Sect. 5.1). Despite the large variability in the number of interactions (notice the log scale), we observe a strong correlation, resulting in a Pearson correlation coefficient of about 0.90 (in physical time) and 0.92 (in virtual time). Similar strong correlations was observed for other influencers, on both IG and FB, and considering different measurement times (e.g., even after just a few minutes after post creation). This result motivated us to develop the model that will be presented later in Sect. 5.

**Fig. 10:**
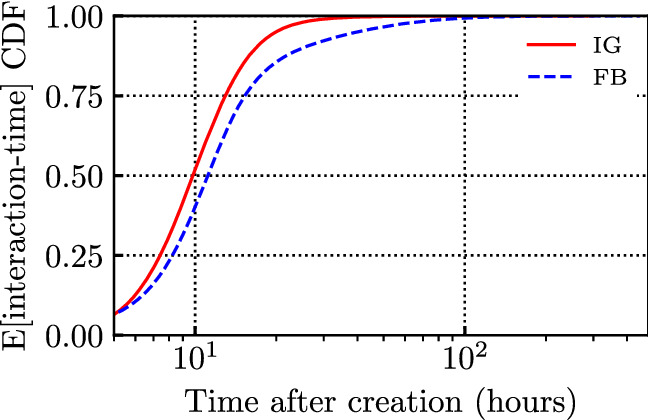
Post mean (expected value) of post interaction-time

In addition, we computed the mean arrival time, defined as the average time after which an interaction occurs, after post creation. The average is computed over 480 h, for a given post of a given influencer, using the empirical distribution of all interaction arrival times. Figure 10 depicts (in log x-scale) the CDF (among different posts of the same influencer) of the mean arrival time of interactions. We consider posts with at least 1,000 interactions and focus on the first 480 h. We can observe that posts on FB are characterised by a higher mean arrival time with respect to IG: 15 h for FB, and 11 h for IG. The faster dynamic in IG confirms what Fig. 7 already suggested.

**Fig. 11:**
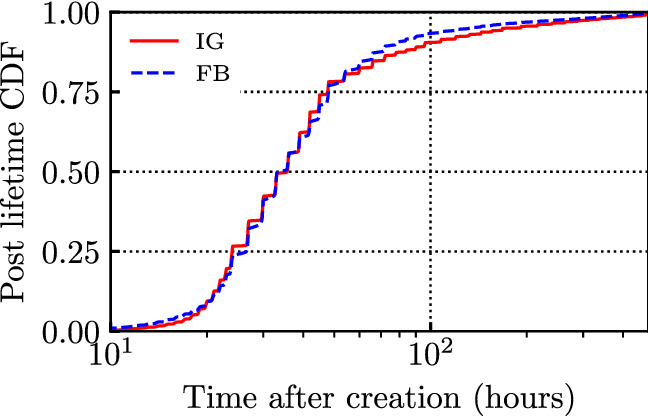
Lifetime of a post computed as 95-th percentile of interactions

Finally, we investigate the lifetime of posts. To this end, we consider that a post basically no longer attracts interactions after 20 days, and thus we define as total number of interactions received by a post the number of interactions received after 20 days. Then, for a given post, we compute its lifetime as the time at which the post has received 95% of its total interactions (as defined above). We consider only posts that collect at least 1000 interactions to get statistically meaningful results. Figure 11 depicts the distribution of the post lifetime in hours, using a log scale on the x-axis; by construction, the maximum lifetime is 480 h, i.e., 20 days. Interestingly, the difference between the two OSNs is small, even though on average FB attracted a smaller fraction of interactions than IG within the first hours (see Fig. 7). The median value of the lifetime is 33 h for both FB and IG, while the mean lifetime is 50 h for FB and 55 for IG.

### Followers’ dynamics

Influencers do not have a constant number of followers over time. Rather, such a number typically increases monotonically over time, with IG exhibiting a more significant increase in the analysed time period (2016–2021) than FB. This is likely because FB is an older OSN, already largely widespread in 2016 (i.e., the first year we monitored).

**Fig. 12:**
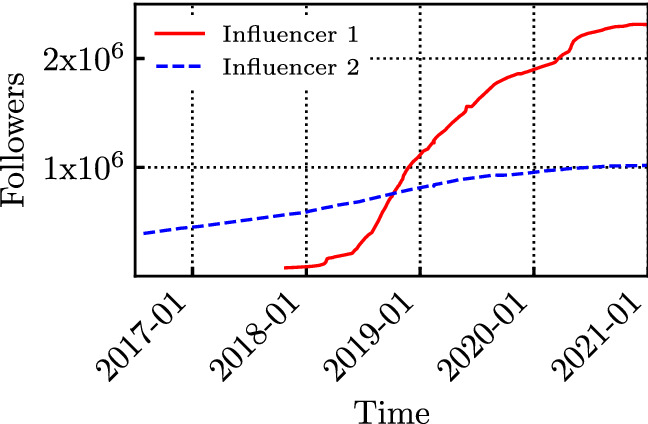
Temporal evolution of the number of followers of Salvini (Influencer 1) and Colombari (Influencer 2) on IG

Figure 12 shows the temporal dynamic of the number of followers for two sample influencers on IG.^5^ Influencer 1 is Matteo Salvini (an Italian politician), while Influencer 2 is Martina Colombari (an Italian actress). Figure 12 suggests that the change in the number of followers can be very different for the influencers. Influencer 1 started using the social network much later (late 2017), and his increase rate varies wildly over time, likely due to reasons unrelated to the operation of the OSN (elections, new laws, etc.). The increase in the number of followers of Influencer 2 is instead smoother over the considered time span.

**Fig. 13:**
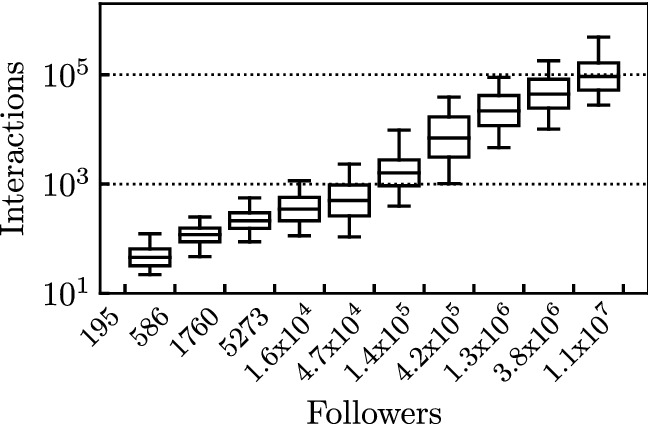
Relation between followers and interactions. Each boxplot represents the distribution of the number of interactions per post, for a given range of the number of followers. Both axes are represented in log scale

Figure 13 shows the distributions of the total number of interactions per post (represented by vertical boxplots), considering all posts published when the number of followers is comprised within the bins specified along the *x* axes (notice that the extremes of the considered bins increase geometrically with ratio $\approx 3$). All posts published on IG available in our dataset are here considered. We notice a strong correlation, suggesting a linear dependence of the mean total number of interactions with the number of followers (we will exploit this dependency in our model in Sect. 5).

**Fig. 14:**
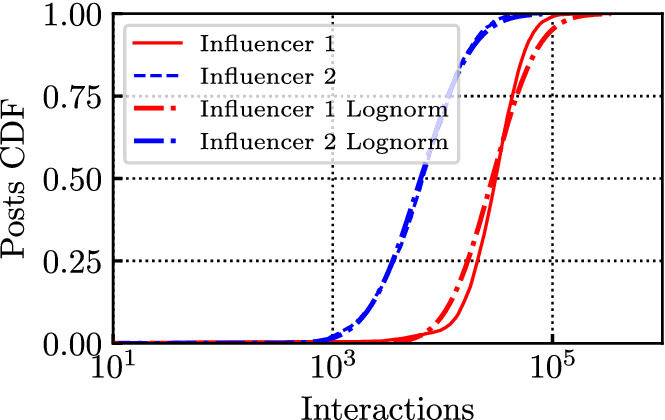
CDF of total number of interactions per post for Salvini (Influencer 1) and Colombari (Influencer 2) on IG at the end of the posts’ lifetime, along with their log-normal fit

**Fig. 15:**
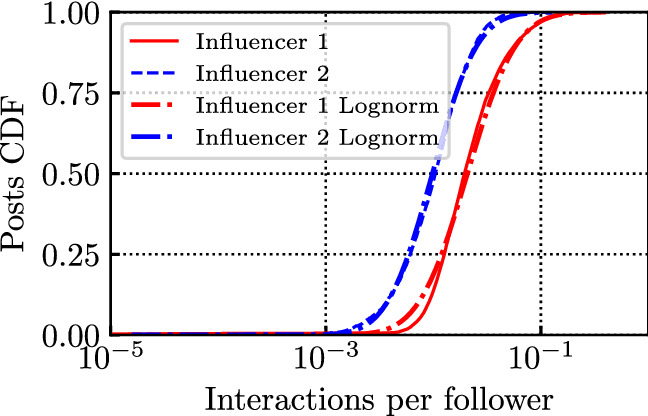
CDF of interactions per follower for Salvini (Influencer 1) and Colombari (Influencer 2) on IG at the end of the posts’ lifetime, along with their log-normal fit

We found that the distribution of the total number of interactions per post is well fit by a log-normal distribution, see the CDFs on Fig. 14. Again, in the figure, we considered the influencers Salvini (Influencer 1) and Colombari (Influencer 2) on IG. Comparing the empirical distribution with the fit, we obtain a Kolmogorov distance of the log-normal of 0.10 and 0.03, respectively for Influencer 1 and 2 (with parameters of the log-normal $\mu =10.2$, $\sigma =0.7$ for Influencer 1 and $\mu =8.7$, $\sigma =0.9$ for Influencer 2). Considering the (almost linear) dependency with the number of followers, as suggested by results in Fig. 13, we also computed a *normalized* total number of interactions, dividing it by the number of followers at each post creation timestamp. We call this number *interactions per follower*. As expected, the log-normal fit is even better when we consider this normalized number, see CDFs in Fig. 15, especially for influencers whose number of followers varies significantly over the considered period (e.g., Influencer 1). Indeed the Kolmogorov distance decreases to 0.05 for Influencer 1, with parameters of the log-normal $\mu =-3.9$, $\sigma =0.8$ (Kolmogorov distance 0.03 for Influencer 2, with parameters $\mu =-4.6$, $\sigma =0.8$). In Appendix 1 we report analogous results related to other kinds of interactions, i.e., shares and comments on FB.

All in all, considering the followers’ dynamics helps to disentangle the impact of the users that potentially interact with the post (i.e., the followers) and the variability of the post intrinsic attractiveness.

### Impact of newly published content

As observed in Sect. 4.2, the arrival rate of new interactions decays roughly exponentially with time. To better understand the nature of the arrival process of interactions generated by a specific post, we asked ourselves whether this is affected by the fact that, meanwhile, new posts are published by the same influencer, thus reducing the ‘novelty’ of the post. For example, Fig. 8a shows a case in which many new posts are published within the first three days after post creation, while in the case of Fig. 8b no new post is published within the first 62 h. On the other hand, after 12 h the first post sample has already collected 91% of its total interactions, while the second post, after the same amount of time, has collected just 62% of its total interactions, due to the fact that its interaction rate decays more slowly. This suggests that the number of newly published content might affect the growth rate of the number of interactions received by a post.

**Fig. 16:**
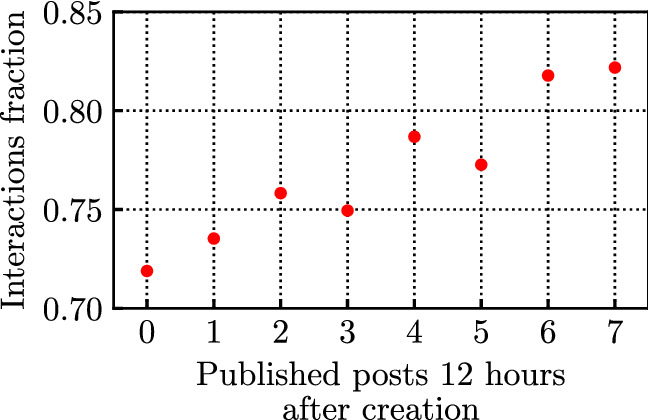
Average fraction of interactions vs no. of published posts, after 12 h since their creation

To verify this, we consider a fixed period of 12 h since the post creation, and compute the number of new posts published within this period. Figure 16 shows the average fraction of interactions collected by a post after 12 h, as a function of the number of newly published posts in the same period, for all posts published by the previously considered influencer Giuseppe Conte. We observe a clear correlation between the two quantities: the higher the number of new posts published within the first 12 h, the faster the post approaches the end of its lifetime. Indeed, in the absence of newly generated posts, a post on average collects 72% of its total interactions within the first 12 h. When 7 newer posts are generated in the same period, the average fraction of collected interactions increases to 82%. This shows that the arrival rate of interactions also depends on how many new posts are published since the post creation, as newly published content can slow down the interaction arrival rate (this can be attributed to the limited budget of attention of users).

## Modelling user interactions

From our measurements and analysis, we learnt several important lessons that can help us model the temporal evolution of the number of interactions collected by a post:

(i) Posts are characterised by an intrinsic initial ‘attractiveness’, which varies significantly even across the posts published by the same influencer;
(ii) The growth rate of interactions naturally decays over time, but the decay rate is itself highly diverse from post to post, besides depending on the considered OSN;
(iii) The interaction rate should be modulated by the daily pattern of user activity, which appears to be independent of the particular online platform;
(iv) On average, there is a linear dependency between the total number of interactions received by a post, and the current number of followers (which can be considered constant during the short post lifetime);
(v) The distribution of the total number of interactions, normalised by the number of followers, is well fit by a log-normal distribution, whose parameters depend on the specific influencer and OSN;
(vi) The generation of new posts by the same influencer progressively reduces a post’s attractiveness level. This can be explained by the fact that users focus their attention on the posts at the top of the timeline.

Despite the intrinsic difficulties in incorporating all of the above features into a simple and tractable model, our preliminary investigation (see Fig. 9) suggests that it is feasible to accurately predict the total number of interactions after observing the very initial phase of the post lifetime. With this objective in mind, we propose the analytical methodology described in the following sections.

### Removal of daily activity effects

Given a trace (i.e., time evolution data) of users interactions $\{t_i\}$, $t_i > 0$ with a given post published at time 0, we can easily derive a modified trace $\{t'_i\}$ in which the impact of variable daily activity is removed. Let $\lambda (t): [0,24]\rightarrow \mathcal{R}^+$ be the daily followers’ activity averaged across all posts of a given influencer (here *t* is in hours), similar to what is shown for all influencers in Fig. 4. Let $\bar{\lambda } = \frac{1}{24} \int _0^{24} \lambda (t) \text {d}t$ be the average user activity across the day.

Assuming that the post was published at hour $T_0 \in [0,24]$, we define the modulating function *g*(*t*), $t \ge 0$, as:

$$\begin{aligned} g(t) = \lambda ((t+T_0)\, \text {mod}\, 24) \end{aligned}$$

which is simply a shifted and replicated version of $\lambda (t)$ providing the expected activity of users at an arbitrary time *t* after the post publication. Then, an interaction which occurred at real time $t_i$ is shifted to virtual time $t'_i$:

1 $$\begin{aligned} t'_i = \frac{\int _0^{t_i} g(t) \text {d}t}{\bar{\lambda }} . \end{aligned}$$

Note that the above transformation preserves the ordering of interactions, i.e., if $t_i > t_j$ then $t'_i > t'_j$, while removing the impact of variable daily activity by diluting (densifying) interactions occurring in periods of high (low) activity.

We expect the virtual trace $\{t'_i\}$ to be more regular than the real trace $\{t_i\}$, and thus easier to model and predict.

At last observe that if $g(\cdot )$ is assumed to be continuous, previous equation can be rewritten as:

$$\begin{aligned} t'_i = t'_{i-1}+ \frac{\int _{t_{i-1}}^{t_i} g(t) \text {d}t}{\bar{\lambda }}= t'_{i-1} + \frac{g(\psi )}{\bar{\lambda }}(t_{i}-t_{i-1}) \end{aligned}$$

for some $\psi \in [t_{i-1},t_{i}]$. In particular, if $g(\cdot )$ is sufficiently slowly varying, we can approximately write:

$$\begin{aligned}t'_{i} \approx t'_{i-1} + \frac{g(t_{i-1})}{\bar{\lambda }}(t_{i}-t_{i-1})1,. \end{aligned}$$

**Fig. 17:**
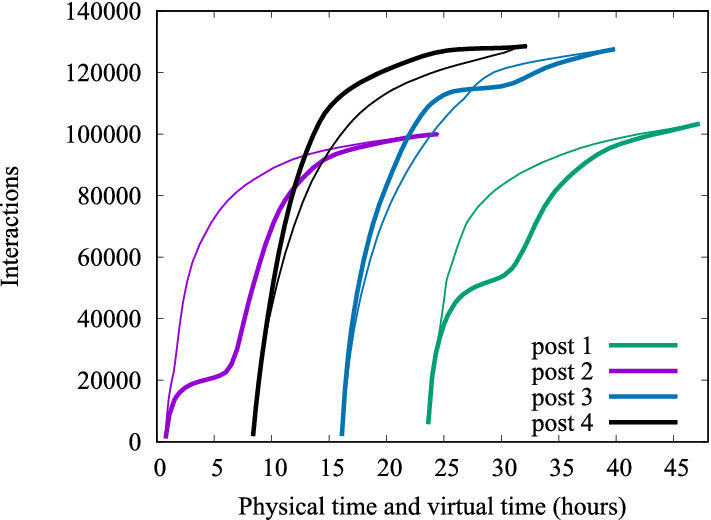
Examples of traces of temporal evolution of interactions over posts (thick curves) transformed into virtual time (thin curves)

Figure 17 shows some examples of traces of the number of interactions accumulated over time by four posts, published roughly at 1am (purple), 8am (black), 4pm (blue), and 12pm (green). Thick curves refer to physical time *t*, while thin curves refer to virtual time $t'$, and were obtained by applying transformation (1). We observe that the virtual time transformation removes the ‘plateau’ due to low user activity late at night (purple and green curves). Similarly, it allows us to distribute more smoothly over time interactions accumulated over periods of high user activity, like at midday (black curve), or early at night (blue curve).

From now on, we will only reason in terms of virtual time, assuming that any measurement $N(T_0,t)$ of the number of interactions collected by a post published at time $T_0$, within time *t*, has passed through transformation (1), producing an equal number $N(T_0,t')$, shifted at virtual time $t'$.

### Modelling the generation of interactions

Let us assume that each post is characterised by an intrinsic level of attractiveness described by a positive real-valued mark $X \in \mathcal{R}^+$. Marks associated with posts of a given influencer are assumed to be i.i.d. with PDF $f_X()$. We can consider some simple law for $f_X()$, incorporating long-tail behaviour, e.g., a log-normal distribution with parameters $\mu _X$, $\sigma _X$.

We assume that the final number $N_\infty$ of interactions received by a post is equal to *F*(0)*X*, where *F*(0) is the number of followers at the time of the post creation. Note that, if $X \sim \text {Lognormal}(\mu _X,\sigma _X^2)$, $F(0) X \sim \text {Lognormal}(\mu _X + \log (F(0)),\sigma _X^2)$.

Let $N(t')$ be the number of interactions received within (virtual) time $t'$ after the post creation. First, we condition on $N_\infty = n_\infty$:

$$\begin{aligned} \!N(t') \!\mid \! (N_\infty \!=\! n_\infty ) = \sum _{i=1}^{n_\infty } {\varvec{1}}\{\text {user }i\text { interacts before }t'\} \end{aligned}$$

where ${\varvec{1}}$ is the indicator function. We assume that followers access the platform (independently from each other) according to a Poisson process of rate $\Lambda$, which is itself a random variable with probability density function $f_\Lambda (\lambda )$. Let $\mathcal{F}_\Lambda (s) = \mathbf {E}[e^{-s \Lambda }]$ be the Laplace transform of $f_\Lambda (\lambda )$. Then:

$$\begin{aligned} {\varvec{1}}\{ \text {user }i\text { interacts before }t' \} \mid \Lambda _i = \lambda \end{aligned}$$

is a Bernoulli random variable with mean $1-e^{-\lambda t'}$. It follows that

2 $$\begin{aligned} \mathbf {E}[N(t') \mid (N_\infty = n_\infty )]= & {} n_\infty \mathbf {E}[1-e^{-\lambda t'}]\nonumber \\= & {} n_\infty (1-\mathcal{F}_\Lambda (t')) \end{aligned}$$

Moreover, we note that

$$\begin{aligned} \mathbf {E}\left[ \frac{N_\infty -N(t')}{N_\infty } \mid (N_\infty = n_\infty )\right] = \mathcal{F}_\Lambda (t') \end{aligned}$$

does not depend on $N_\infty$; hence, we can obtain the Laplace transform of $f_\Lambda (\lambda )$ by averaging out $N_\infty$:

3 $$\begin{aligned} \mathcal{F}_\Lambda (t') = \mathbf {E}\left[ \frac{N_\infty -N(t')}{N_\infty }\,\right] . \end{aligned}$$

We found empirically that a surprisingly accurate model for $f_\Lambda (\lambda )$ is a mixture of a uniform distribution in [0, *a*] and an exponential distribution of parameter $\delta$:

$$\begin{aligned} f_\Lambda (\lambda ) = m \frac{1}{a} {\varvec{1}}\{\lambda < a\} + (1-m) \delta e^{-\delta \lambda } \end{aligned}$$

from which

$$\begin{aligned} \mathcal{F}_\Lambda (s) = m \frac{1-e^{-a\,s}}{a\,s} + (1-m) \frac{\delta }{s+\delta } . \end{aligned}$$

**Fig. 18:**
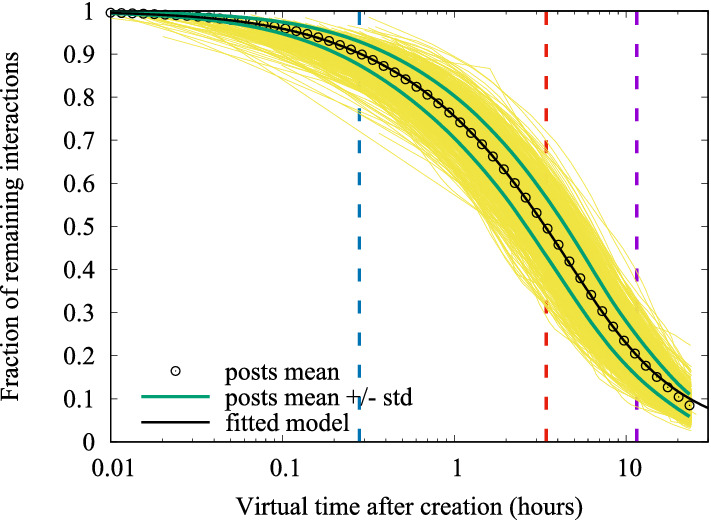
Fitted Laplace transform of user access rate $\lambda$

Parameters *m*, *a*, $\delta$ have to be fitted for each specific influencer, though they do not vary significantly from influencer to influencer, as shown in the following.

Figure 18 presents the fitted Laplace transform $\mathcal{F}_\Lambda (s)$, through parameters *m*, *a*, $\delta$, using the traces of 9,204 posts published by Italian politician Matteo Salvini on IG. Fitted values are: $m = 0.83$, $a = 0.41$, $\delta = 0.7$. Figure 18 requires a careful explanation. First of all, notice the log *x* axes, spanning from 0.01 hour (36 s) to 24 hours. Since in the following we will be especially interested in the early stages of post lifetime, this will be the time scale used in all plots hereinafter.

The vertical axes reports the fraction of residual interactions, $\frac{N_\infty -N(t')}{N_\infty }$. By (3), the mean across all traces of this fraction provides the sought Laplace transform $\mathcal{F}_\Lambda (s)$ for Influencer Salvini. Small circles show such averaged fraction at various points in time, while the black solid curve is the fitted model, which turns out to be very accurate. The figure also shows the ensemble of 1000 actual traces (in yellow), which produces a large band around the mean. At last, green curves above and below the mean are plotted at a distance equal to the measured standard deviation.

**Fig. 19:**
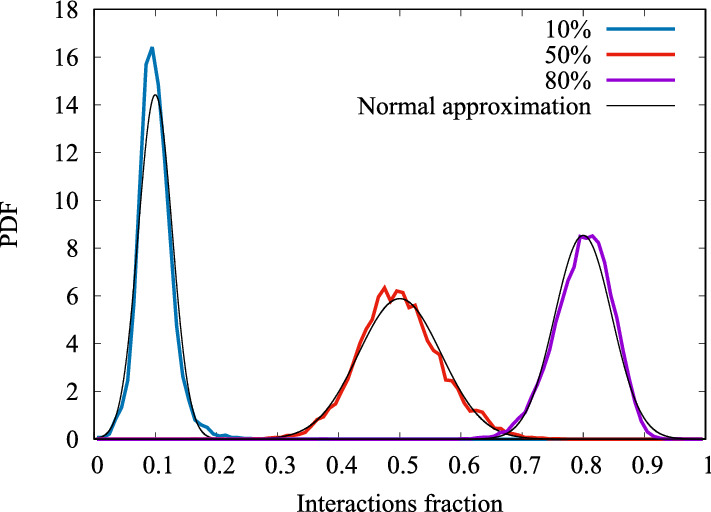
Empirical PDF of the fraction of arrived interactions, at the time instants when the mean fraction of arrived interactions is equal to 10%, 50%, 80%. Black curves show the fit obtained by a normal distribution

Figure 18 reveals that there is a significant variability of traces around the mean, which is, unfortunately, not captured by the model introduced so far.^6^ However, we found that the distribution of the fraction of residual interactions (similarly, the distribution of the fraction of already collected interactions) is approximately normal. This fact is shown in Fig. 19, which depicts the empirical distributions of the fraction of received interactions, measured at the times at which the mean fraction of received interactions is equal to 10% (blue), 50% (red), 80% (purple), as denoted by vertical dashed lines in Fig. 18.

Note that the mean fraction of collected interactions is given by

4 $$\begin{aligned} \mu (t') = 1 - \mathcal{F}_\Lambda (t') \end{aligned}$$

for which we already have an accurate model. However, we still lack a model providing the deviation $\sigma (t')$. We suspect that, beyond the initial level of attractiveness *X*, the post dynamics is characterised by random temporal fluctuations of the rate at which users interact with it. These fluctuations are due to time-varying popularity, generation of new posts (which tends to decrease the attention of users on the considered post, see Sect. 4.4), and self-reinforcement effects due to users observing the engagement of other users (which can increase the interaction rate after a period of low user interest).

In order to incorporate the effects all such elements in the model, we resorted to a simple fitting of the empirical standard deviation by a 2-parameter curve. Specifically, we found that the function:

5 $$\begin{aligned} \sigma (t') = c\, t'^b e^{-\sqrt{t'}} \end{aligned}$$

provides a reasonable approximation, where parameters *c* and *b* can be computed for each influencer, though they do not vary significantly from influencer to influencer (however, we noticed that traces on FB have larger variability than traces on IG, see Tables 2 and 3).

**Fig. 20:**
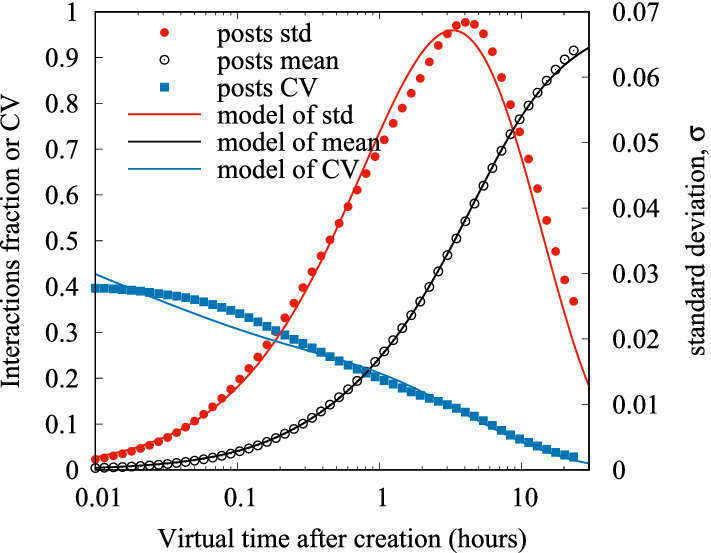
Fitted models for the mean (left y axes) and standard deviation (right y axes) of the fraction of collected interactions. The resulting coefficient of variation is also shown (left y axes)

Figure 20 shows the empirical standard deviation of traces of influencer Salvini on IG (red circles), and the best fit by the proposed function (5) (solid red curve). It also repeats the fit for the mean already shown in Fig. 17, but this time in terms of average fraction $\mu$ of already received interactions (black). Indeed, what is ultimately important is to obtain a good estimate of the coefficient of variation $CV = \sigma /\mu$, which is also shown on the plot (blue).

Knowing that the fraction of received interactions within time $t'$ is approximately normal, and having derived parameters $\mu (t')$ and $\sigma (t')$ as function of (virtual) time $t'$ (for each influencer and social platform), we can now make analytical predictions of post dynamics.

For example, in Table 2 we report some predictions obtained for six different influencers on IG (first column). The other columns provide, from left to right: the average fraction of interactions collected during the first 6 min, and the corresponding standard deviation; the average fraction of interactions collected during the first hour, and the associated standard deviation; the time at which we expect to see half of the total interactions, denoted as $t(50\%)$; the time at which we expect to see 80% of total interactions, denoted as $t(80\%)$; the maximum standard deviation over all time (denoted by ${\sigma }_{\max }$). We report the values observed from the collected data and the corresponding values obtained from the analytical model for each influencer.

Table 3 reports similar results for six influencers on FB. Besides noticing the good fit of the model in all cases, it is interesting to see that some numbers are surprisingly similar across different influencers and platforms: roughly 4% of all interactions are collected within 6 min since post creation, and roughly 25% after one hour; on IG, 50% (80%) of all interactions are collected after roughly 3.3 (12.4) hours; on FB, these last figures are a bit larger: 50% (80%) of all interactions are received after roughly 3.8 (17) hours.

**Fig. 21:**
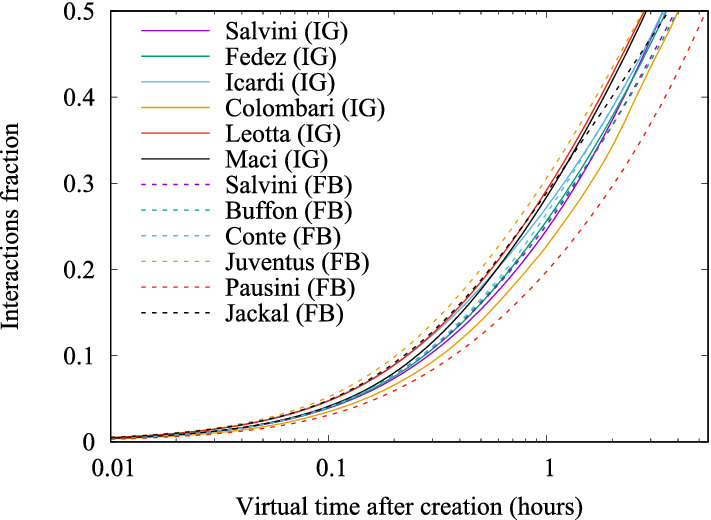
Average fraction of collected interactions as function of time, for various Influencers (from Tables 2 and 3)

The similarity of results for the mean fraction of collected interactions is further illustrated in Fig. 21, showing on the same plot the curves $\mu (t') = 1 - \mathcal{F}_\Lambda (t')$ computed analytically for all 12 influencers considered in Tables 2 and 3.

At last, as anticipated, it is interesting to observe (last column of the tables) that the maximum standard deviation of the traces is larger on FB than on IG, by a factor of about 1.5.

**Table 2:** Results for various influencers, comparing observed behaviour and model output (IG)

Influencer	# Traces		Interaction fraction after 6 min		Interaction fraction after 60 min		$t(50\%)$	$t(80\%)$	$\sigma _{\max }$
			Avg	Std	Avg	Std			
Salvini (politician)	9204	Observed:	0.039	0.013	0.246	0.049	3.4	11.5	0.068
		Model:	0.041	0.012	0.244	0.051	3.3	11.6	0.067
Fedez (singer)	1988	Observed:	0.040	0.019	0.257	0.064	3.5	12.8	0.104
		Model:	0.042	0.017	0.254	0.070	3.4	13.0	0.094
Icardi (sportsman)	1714	Observed:	0.048	0.016	0.272	0.048	3.4	13.6	0.069
		Model:	0.050	0.015	0.271	0.053	3.3	13.0	0.065
Leotta (presenter)	1280	Observed:	0.047	0.018	0.292	0.052	2.8	11.0	0.077
		Model:	0.048	0.010	0.292	0.052	2.7	11.0	0.077
Colombari (actress)	889	Observed:	0.035	0.035	0.227	0.090	4.0	15.1	0.128
		Model:	0.035	0.028	0.228	0.101	4.0	14.8	0.124
Maci (food-blogger)	814	Observed:	0.041	0.013	0.284	0.056	2.8	10.3	0.084
		Model:	0.045	0.013	0.280	0.059	2.8	10.4	0.080

**Table 3:** Results for various influencers, comparing observed behaviour and model output (FB)

Influencer	# Traces		Interaction fraction after 6 min		Interaction fraction after 60 min		$t(50\%)$	$t(80\%)$	$\sigma _{\max }$
			Avg	Std	Avg	Std			
Salvini (politician)	10,243	Observed:	0.040	0.025	0.252	0.098	3.9	16.1	0.124
		Model:	0.041	0.024	0.252	0.102	3.9	16.1	0.135
Juventus (sport club)	3226	Observed:	0.052	0.039	0.306	0.124	2.7	12.9	0.143
		Model:	0.053	0.036	0.308	0.132	2.7	12.8	0.163
Conte (politician)	789	Observed:	0.039	0.024	0.265	0.104	3.5	16.3	0.137
		Model:	0.041	0.023	0.265	0.106	3.5	16.1	0.146
Pausini (singer)	539	Observed:	0.031	0.016	0.198	0.071	5.3	19.4	0.112
		Model:	0.032	0.015	0.197	0.075	5.4	19.8	0.109
Jackal (comedians)	417	Observed:	0.048	0.045	0.289	0.157	3.6	23.2	0.212
		Model	0.048	0.043	0.289	0.168	3.5	23.4	0.214
Buffon (sportsman)	349	Observed:	0.040	0.017	0.255	0.064	3.9	14.5	0.085
		Model:	0.044	0.016	0.251	0.067	3.8	14.9	0.088

### Model exploitation: post popularity prediction

**Fig. 22:**
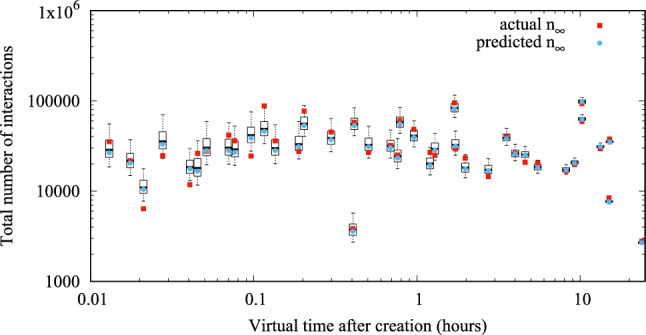
Boxplots of a-posteriori distributions of $N_\infty \mid n(t')$, predicted value $\hat{n}_\infty$ (circles), actual values $n_\infty$ (squares), for 40 different posts of Influencer Salvini on IG, starting from (single) observations $n(t')$, where $t'$ is reported on the *x* axes

One of the most interesting applications of our model is the early prediction of post popularity, which can have several applications. For example, the social platform can use this prediction to sell advertisement slots to be shown in proximity of the post, and prediction of the number of views that the post will receive in the future is crucial to bid a price for the available ad slots.

Suppose to measure the number of interactions *n*(*t*) received by a post, published at time $T_0$ by a given influencer, after a period of duration *t*. What can we infer about the total number of interactions $n_\infty$ that the post will eventually receive?

Our analysis suggests the following approach should be taken. First, suppose to know the number of followers *F*(0) at the post creation time. Moreover, assume that analysis of the history of post popularity of the given influencer has allowed us to estimate parameters $\mu _X$, $\sigma _X$ of the log-normal distribution of the intrinsic level of attractiveness *X* (see Sect. 4.3). Then the *unconditioned* distribution of the random variable $N_\infty$ is $\text {Lognormal}(\mu _X + \log (F(0)),\sigma _X^2)$.

A standard maximum a posteriori estimation (MAP) allows us to compute a prediction $\hat{n}_\infty$ on the total number of interactions that the post will receive, *given* observation *n*(*t*). First, we transform the observation *n*(*t*) into virtual time $n(t')$ to remove the effect of daily variation of user activity. This is an important step: for example, if a post is published late at night, it might eventually become popular even if it receives just a few interactions during, say, the first hour.

We assume that analysis of the history of the post dynamics of the given influencer has allowed us to fit parameters of functions $\mu (t')$ (4) and $\sigma (t')$ (5). Then the conditioned distribution of random variable $N(t')\mid n_{\infty }$ is normal $\mathcal{N}(n_{\infty } \mu (t'), n^2_{\infty } \sigma (t')^2)$. A standard application of Bayes’ theorem provides the posterior distribution of $N_\infty \mid n(t')$:

6 $$\begin{aligned} \mathbf {P}[ N_\infty= & {} n_\infty \mid n(t') ]\nonumber \\= & {} \frac{ \mathbf {P}[N(t') = n(t') \mid n_\infty ] \mathbf {P}[n_\infty ]}{\sum _{n'_\infty } \mathbf {P}[N(t') = n(t') \mid n'_\infty ] \mathbf {P}[n'_\infty ]} \end{aligned}$$

and our MAP prediction will be the mode of it:

7 $$\begin{aligned} \hat{n}_\infty (n(t')) = \mathop {\mathrm {arg\,max}}\limits _{n_\infty } \mathbf {P}[ N_\infty = n_\infty \mid n(t') ] . \end{aligned}$$

Note that the above analysis also provides an estimate of the error that we will run into by our prediction, since we have the entire posterior distribution of $N_\infty \mid n(t')$.

As an example, Fig. 22 shows the MAP prediction (blue circles) of $N_\infty$ for 40 posts published by influencer Salvini on IG, given some observation $n(t')$ (one for each post), where $t'$ is shown on the horizontal axes. Red squares denote true values of $N_\infty$, while boxplots provide a graphical representation of the posterior distribution of $N_\infty \mid n(t')$ computed analytically. We can observe from these sampled posts that the larger the time $t'$ at which we observe the number of interactions, the smaller the prediction error in the total number of interactions, as expected. However, Fig. 22 suggests that accurate predictions are already feasible a short time after post creation.

#### Comparison with baseline model

To better show the goodness of our approach, we compare our predictions with those obtained by a baseline model. In this baseline model, followers of a given influencer independently access the platform according to a Poisson process of rate $\lambda _a$, where $\lambda _a$ is the *same* for all users. Moreover, suppose that the decision to interact with a given post is made independently from the access time to the platform, and independently from user to user. Consequently, followers who decide to interact with a given post will do so after an amount of time distributed according to an exponential distribution of parameter $\lambda ^*$, where $\lambda ^*$ is the *same* for all users interacting with a given post.

For a fair comparison with our model, we will assume that the baseline model shares the same information about the history of posts of a given influencer. In particular, the distribution of the final number of interactions received by a post is known, modelled by a fitted $\text {Lognormal}(\mu _X + \log (F(0)),\sigma _X^2)$, where *F*(0) is the number of followers at the time of the post creation (see Sect. 5.2). Moreover, we assume that detailed temporal history of interactions allows the baseline model to fit its single parameter $\lambda ^*$ against the trace of all posts generated by a given influencer (i.e., $\lambda ^*$ is adapted to each specific influencer). Finally, again for the sake of a fair comparison, the baseline model is applied to the temporal evolution of interactions transformed into virtual time to remove daily effects.

One can easily see that our model subsumes the above baseline model, when $f_\Lambda (\lambda ) = \delta (\lambda - \lambda ^*)$, where $\delta ()$ is Dirac’s Delta function. Its Laplace transform $\mathcal{F}_\Lambda (s) = e^{-s \lambda ^*}$ can then be fitted against the normalized traces of the residual number of interactions, as illustrated in Fig. 18.

Following the same MAP framework introduced before, let $n_\infty$ be an instance of the final number of interactions received by a post, and $n(t')$ be the number of interactions observed after virtual time $t'$ since post creation. Notice that, according to the baseline model, the conditioned distribution of random variable $N(t')\mid n_{\infty }$ can be approximated by a normal $\mathcal{N}(n_{\infty } q(t'), n_{\infty } q(t')(1-q(t')))$, where $q(t') = 1-e^{-\lambda ^* t'}$, being the sum of $n_\infty$ independent Bernoulli random variables of mean $q(t')$. Therefore, we can apply (6) as well to the baseline model and compute a MAP prediction for the final number of interactions according to (7).

**Fig. 23:**
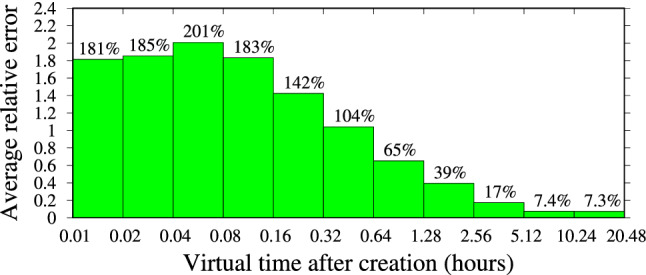
Baseline model: average relative error in the prediction of the total number of interactions received by 1000 posts (for each bin) of influencer Salvini on IG

Figure 23 shows the average relative error of the baseline model in the prediction of $N_\infty \mid n(t')$, for influencer Salvini on IG, considering 1,000 posts for each bin, i.e., for each bin in Fig. 23, we have averaged the relative error $|\frac{\hat{n}_\infty - n_\infty }{n_\infty }|$ of 1,000 different posts (for which an observation $n(t')$ is available in the dataset such that $t'$ falls in the bin).

**Fig. 24:**
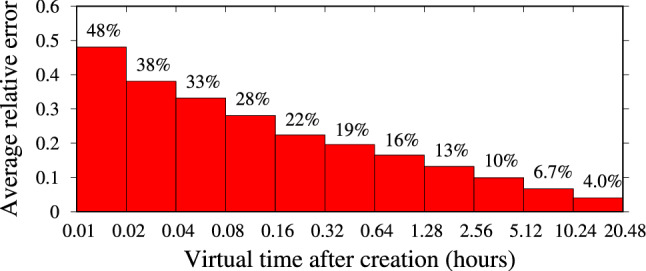
Our model: average relative error in the prediction of the total number of interactions received by 1000 posts (for each bin) of influencer Salvini on IG

In contrast, Fig. 24 shows corresponding results obtained with our approach. We observe a significant reduction in the prediction error as obtained by our model, with respect to the baseline model, especially for smaller values of the measurement time $t'$, suggesting that our approach is significantly better at performing early prediction of the final number of interactions. As expected, the relative error of the prediction diminishes over time. It is remarkable that a relative error of only 48% is incurred if an observation is available just between 0.01 (36 s) and 0.02 (72 s) after the post creation, i.e., a very early prediction. After 6 min (0.1), the error reduces to about 28%, and after about 1 h it reduces to about 16%. Similar results, not shown here, for the sake of brevity, have been obtained for the other considered influencers.

The superiority of our approach is essentially due to the fact that the baseline model describes a homogeneous population of followers through a single parameter ($\lambda ^*$). In contrast, our model employs multiple parameters to describe heterogeneous followers, accounting for the fact that different users interact with posts more or less promptly, depending on the frequency with which they access the platform, which is highly diverse from user to user.

## Discussion and conclusion

In this work, we studied the temporal dynamics of Facebook and Instagram for five years, focusing on top Italian influencers. After a thorough analysis of real-world data, we characterised several interesting features of the above OSNs, including: (i) the influencers’ and followers’ activity over time, (ii) the posts inter-arrival time and the post life-time, (iii) the arrival process of user interactions with a given post. The insights gained from our dataset analysis allowed us to develop a mathematically tractable, yet accurate model describing the temporal evolution of the number of interactions collected by a post. We validated our model against real traces for both Facebook and Instagram. In particular, we demonstrate our model’s ability to perform early prediction of post popularity and the large improvements with respect to a simpler baseline. The existence of many interesting possible applications that may profit from early popularity predictions, such as anomaly detection and price bidding of ad slots, encourages further analytical efforts in this direction to incorporate effects not yet captured by the proposed methodology.

## Figures and Tables

**Fig. 25:**
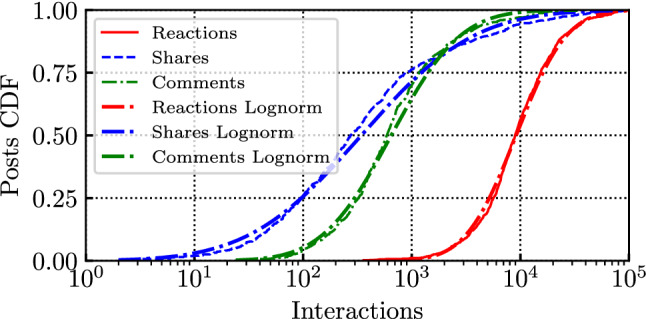
CDF of the total number of interactions, measured as reactions, shares, and comments on influencer Jackal (comedians) on FB at the end of the posts’ lifetime, along with their log-normal fit

**Fig. 26:**
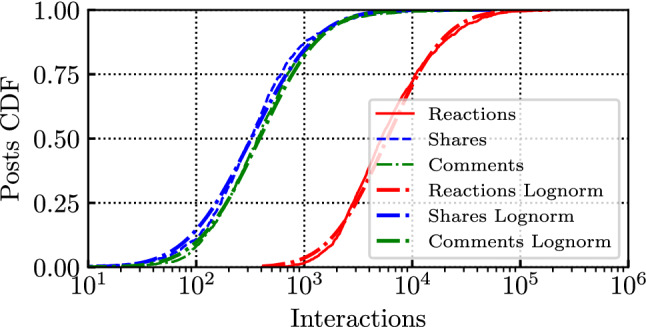
CDF of the total number of interactions, measured as reactions, shares, and comments on influencer Laura Pausini (singer) on FB at the end of the posts’ lifetime, along with their log-normal fit

**Fig. 27:**
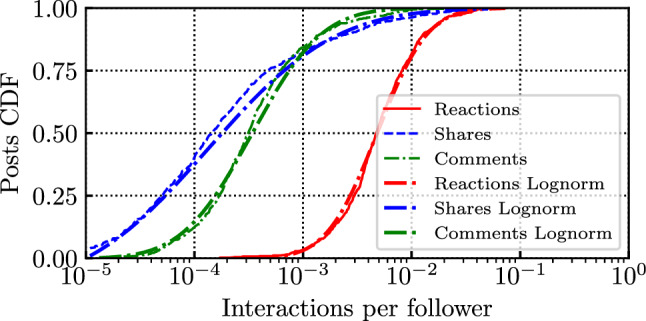
CDF of the total number of interactions per follower (measured as reactions, shares, and comments per follower) on influencer Jackal (comedians) on FB at the end of the posts’ lifetime, along with their log-normal fit

**Fig. 28:**
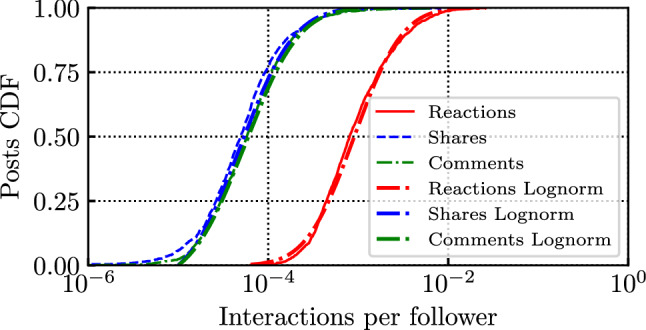
CDF of the total number of interactions per follower (measured as reactions, shares, and comments per follower) on influencer Laura Pausini (singer) on FB at the end of the posts’ lifetime, along with their log-normal fit

## Appendix 1. Shares, comments, and reactions on FB

The interactions with an influencer’s post can be measured in different ways: in this paper, we focus on the total number of likes/reactions, but this metric can be complemented, or substituted, by the number of shares of the post and the number of comments (see Table 1).

On FB, a user can share an influencer’s post, and this action will appear as a new post from the user. In Fig. 14 we presented the CDFs of the total number of likes for two influencers of IG, and we showed the goodness of fit with a log-normal distribution. Here, we repeat the analysis by also considering the number of shares and comments as different metrics for interactions on Facebook. Figures 25 and 26 depict such different metrics, along with their fittings, for two of the studied influencers of FB, namely Jackal, a comedian group, and Laura Pausini, a singer (see Table 3). As can be seen, most interactions consist of reactions, while the number of comments and shares is at least an order of magnitude smaller. However, the behaviour of these metrics is again well approximated by a log-normal distribution. Regarding the first influencer (Fig. 25), the Kolmogorov distance of the log-normal is 0.04 for reactions, 0.06 for shares, and 0.07 for comments. For the second example influencer (Fig. 26), the Kolmogorov distance is 0.05 for reactions, 0.06 for shares, and 0.06 for comments.

Similarly to Fig. 15, we also report the normalised total number of interactions, dividing them by the number of followers at each post creation timestamp. Again, we analysed reactions, shares, and comments. The results are presented in Figs. 27 and 28, respectively for influencer Jackal, and influencer Laura Pausini. As for the examples on IG, the log-normal fit is as good (or even better) when considering this normalized number.

In conclusion, shares and comments follow a distribution shape similar to the one of reactions, and they are well fitted by a log-normal distribution, even though their absolute number is smaller.

## Footnotes

1 https://smartdata.polito.it/temporal-dynamics-social/.

2 https://github.com/CrowdTangle/API.

3 https://www.facebook.com/formedia/tools/crowdtangle.

4 Due to limited granularity of available measurements, we considered time instants in the range [0.45, 0.55] hours after post creation.

5 We do not report analogous results for FB since, according to CrowdTangle, “Follower data prior to September 16, 2020, is incomplete and does not include either unfollows or Page-to-Page follows”.

6 The variance calculated according to the proposed model would be negligible.
